# Second‐order elasticities for ecology and evolution: Unraveling nonlinear fitness responses to perturbations

**DOI:** 10.1002/ecy.70479

**Published:** 2026-09-11

**Authors:** Maja Kajin, Shripad Tuljapurkar, Wenyun Zuo, Harman Jaggi, Samuel Gascoigne, Roberto Salguero‐Gómez

**Affiliations:** ^1^ Department of Biology, Biotechnical Faculty University of Ljubljana Ljubljana Slovenia; ^2^ Department of Biology University of Oxford Oxford UK; ^3^ Department of Biology Stanford University Stanford California USA; ^4^ Department of Ecology and Evolutionary Biology Princeton University Princeton New Jersey USA; ^5^ School of Biological Sciences, University of Aberdeen Aberdeen UK

**Keywords:** diminishing or accelerating returns, elasticity, life history evolution, marginal rate of substitution, mean individual fitness, second‐order derivative, trade‐off, transient‐response matrix

## Abstract

In ecology and evolutionary biology, understanding the relationship between vital rates (e.g., survival, development, reproduction) and population growth is essential to elucidate how life history strategies are shaped by natural selection. Yet most established demographic methods rely on linear assumptions that rank the importance of vital rates but are insensitive to whether the returns of the population growth rate are diminishing or accelerating. Here, we introduce the second‐order elasticities of mean individual fitness in the population, the S‐elasticity. The S‐elasticity is a proportional, element‐wise measure that can quantify the curvature of mean individual fitness with respect to single matrix population model (MPM) elements (self S‐elasticity) and to pairs of elements (mixed S‐elasticity). S‐elasticities (1) classify concavity versus convexity of local fitness surfaces, (2) provide a relative‐curvature diagnostic by scaling second‐order to first‐order effects, (3) sum to zero across elements—facilitating comparisons across populations and species—and (4) connect fitness curvature to biologically feasible trade‐offs via the elasticity ratio *K*. We provide a simple inequality based on the S‐elasticities and the *K*‐ratio that detects local fitness peaks along constraint directions corresponding to each pair of matrix elements. We illustrate the framework with an average 4 × 4 MPM for *Didelphis aurita* (Marsupialia, Didelphidae) and compare our results to classical second‐order derivatives of population growth rate applied to the same dataset. First‐order elasticities identify survival of weaned and subadult survival as dominant linear levers of fitness; their negative self S‐elasticities reveal diminishing returns for increases and amplified losses for decreases relative to linear expectations. Mixed S‐elasticities expose a costly trade‐off between survival of weaned and aged females' fertility: The curvature‐only surface is concave near the baseline and the trade‐off direction is a local peak, whereas several other element pairs lie along flatter, more substitutable directions. Together, S‐elasticities provide a compact diagnostic for when linear rankings suffice, when nonlinearity dominates, and where feasible substitutions are most constrained. Because they are proportional, additive (sum‐to‐zero), and rooted in life cycle pathways, S‐elasticities complement classic elasticity and make second‐order demographic information directly comparable across systems—yielding actionable guidance for evolutionary inference and for prioritizing management actions under realistic trade‐offs.

## INTRODUCTION

In ecological and evolutionary dynamics, the various ways organisms go about completing their life cycles have produced a vast variation of life history strategies (Roff, 2002; Stearns, 1992). Said strategies have evolved over evolutionary time via natural selection (Gadgil & Bossert, 1970), with environmental pressures having shaped how organisms optimize their vital rates of survival, growth, and reproduction (McDonald et al., 2017; Morris & Doak, 2004). As such, life history strategies represent the evolutionary responses of organisms to their respective environments (Hutchings, 2021). However, these life history strategies are not static and continue to be subject to evolution today (Chase, 1999; Jaggi et al., 2024). For instance, studies on guppies (*Poecilia reticulata*) have demonstrated rapid evolutionary changes in generation time, a dominant life history trait (Bassar et al., 2016; Gaillard et al., 1989; Salguero‐Gómez et al., 2016), in response to predation pressure (Reznick & Endler, 1982). Current and projected increases in environmental stochasticity (Alexander et al., 2006; Boyce et al., 2006) present a challenge to life histories that may have evolved under different environmental regimes (Cayuela et al., 2015; Gascoigne et al., 2025; Paniw et al., 2018). Thus, it is important to examine the evolutionary trajectories that have shaped these strategies and mitigate the contemporary pressures that may perturb them from their optimal states.

Life history strategies are governed by how vital rate values change across life cycle stages (Caswell, 2001; Ebert, 1999; Roff, 2002). The stages (i.e., distinct phases that an individual passes through from birth to death) can be defined by age, size, and/or development (Ebert, 1999). The ecological, evolutionary, and conservation biology consequences of changes in life histories have been widely studied by examining the effects of small changes in vital rates (Zuidema & Franco, 2001) using structured population models like the matrix population models (MPMs, Caswell, 1978; Caswell et al., 1984; de Kroon et al., 1986; Crouse et al., 1987; van Tienderen, 1995). The effect of such small changes (or perturbations) on the overall growth rate of the MPM is often described by its elasticity (de Kroon et al., 1986). The growth rate of a population determines the average fitness of individuals in the population (Fisher, 1930). This metric of the *mean individual fitness in the population* (fitness, hereafter) is shaped by the multiple matrix elements (*a*_*i,j*_, where *i* and *j* denote, respectively, the stage contributed to and the stage contributing from in one time step) contained in an MPM. In this context, the usual elasticity measures the linear proportional response of fitness to a small proportional change in those matrix elements (Caswell, 2001; de Kroon et al., 1986; Ebert, 1999; Franco & Silvertown, 2004). Note that while vital rates represent direct biological quantities measured in the field (survival, growth, fecundity, etc.), matrix elements are the combination of one or more vital rates that describe the contribution from one stage to another (e.g., survival × growth). Demographic processes, on the other hand, denote the biological interpretation of the matrix elements (e.g., juvenile survival, probability of transitioning to subadults, adult fertility).

Determining which vital rate has the highest impact on population growth rate has kept conservation biologists busy for decades. The focus on the most impacting vital rate was motivated by the application of elasticity measures to MPMs to optimize management and harvest (Olmsted & Alvarez‐Buylla, 1995), conservation strategies (Heppell, 1998; Steen & Erikstad, 1996; Wisdom et al., 2000), and pest control (McEvoy & Coombs, 1999). Elasticity of population growth rate with respect to vital rates or matrix elements quantifies the instantaneous proportional slope of population growth rate with respect to a small change in the respective vital rate (Caswell, 2001; de Kroon et al., 1986; Ebert, 1999).

Because the long‐term population growth rate is a good proxy for the average individual‐level fitness (Fisher, 1930), elasticities represent a cohesive connection between ecology and evolution (Takada & Shefferson, 2018). This connection arises because elasticities are closely related to selection gradients (de Kroon et al., 1986; Lande, 1982; van Tienderen, 2000), since vital rates can be treated as traits subject to selection (Roff, 2002; Stearns, 1992). Selection gradients (Lande, 1982), quantitatively expressed as the derivative of the fitness function with respect to a trait, indicate how fitness varies linearly with trait values. As such, elasticities can represent a measure of the strength of natural selection acting on each vital rate (Takada & Shefferson, 2018). This dichotomy of their interpretation—the ecological and evolutionary meanings of elasticity—places elasticities in a crossroad: that of eco‐evolutionary dynamics (Shefferson & Salguero‐Gómez, 2015).

**FIGURE 1:**
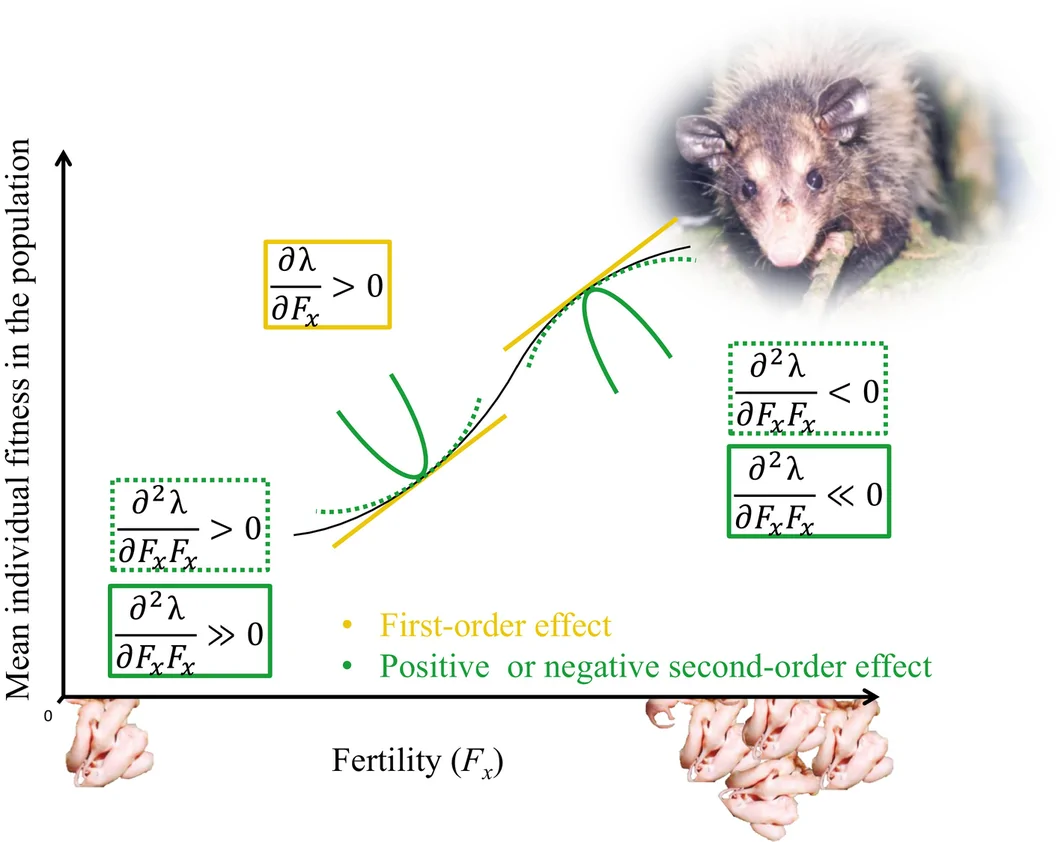
Conceptual fitness curve (black) showing first‐order (yellow) versus second‐order (green) responses to a small change in fertility $F_{x}$ ($F_{x}$ = number of neonates × probability of surviving, symbolized by the number of young opossums inside mother's marsupium on the *x* axis), for *Didelphis aurita*. The first‐order effect is the local slope (elasticity), whereas the second‐order effect is the local curvature (self S‐elasticity): Negative curvature indicates diminishing returns (concavity) and positive curvature indicates accelerating returns (convexity). The figure illustrates concepts; exact forms are given in Equations (1) and (2). Photographs by Maja Kajin.

There is yet another interpretation of elasticities. Because elasticities can be obtained as partial derivatives of the population growth rate (Caswell, 2001; Ebert, 1999), their value represents the rate of change of fitness for the value of a specific demographic process (Figure 1), and they represent scaled sensitivities. For instance, if the first‐order partial derivative of the population growth rate with respect to a MPM element $a_{\mathrm{ij}}$ is positive ($\frac{{\partial \lambda }_{0}}{\partial a_{\mathrm{ij}}}>0$), then fitness is expected to *increase linearly* as the mean value of $a_{\mathrm{ij}}$ across time increases, and vice versa: if $\frac{{\partial \lambda }_{0}}{\partial a_{\mathrm{ij}}}<0$, then fitness is expected to *decrease linearly* as the $a_{\mathrm{ij}}$ mean increases. If $\frac{{\partial \lambda }_{0}}{\partial a_{\mathrm{ij}}}\approx 0$, then changes in the mean of $a_{\mathrm{ij}}$ have no or little effect on fitness. Because elasticities are directly related to the first‐order derivatives, we refer to elasticities as the *first‐order effect on fitness*.

One can also describe a *second‐order effect* of a small change in a matrix element of interest on fitness (Caswell, 2001; Shyu & Caswell, 2014). A second‐order effect also measures the rate of change of fitness at a specific matrix element similarly to a first‐order effect. However, a second‐order effect goes beyond the linearity assumption, revealing the *nonlinearity* of the fitness: whether fitness is *concave* or *convex* with respect to one or more matrix elements. Knowing whether vital rate perturbations produce diminishing versus accelerating fitness returns can shed light on how the variability of that vital rate is harmful or beneficial, on whether selection is stabilizing or disrupting the variance of the population growth rate, and whether management interventions have accelerating or diminishing effects.

### Historical development of perturbation analysis and the resulting implications for evolutionary demography

**FIGURE 2:**
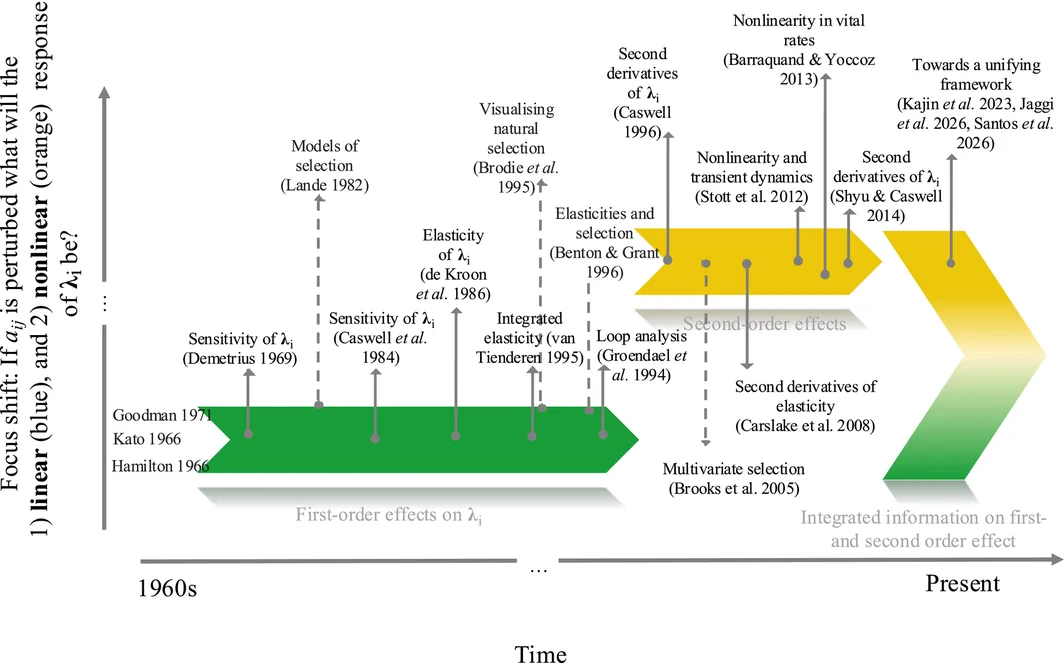
A non‐comprehensive timeline of key perturbation‐analysis papers, highlighting the shift from first‐order (green) to explicit second‐order/nonlinear analyses (yellow) and studies bridging ecological and evolutionary interpretations (dotted gray arrows).

When perturbation‐analysis methods started to be applied in population ecology, the focus was entirely on the first‐order derivatives of population growth rate (Figure 2). Hamilton (1966) famously examined the evolution of senescence in different demographic processes by obtaining the first derivatives of population growth rate (λ) with respect to each demographic process through the characteristic equation derived from the Euler‐Lotka equation (Roff, 2002; Stearns, 1992), similar to Goodman (1971). Kato (1966) provided a rich background of the mathematical machinery behind perturbation analyses, which serves as a general guide in many scientific fields including population ecology (Caswell, 2001; de Kroon et al., 1986; Ebert, 1999). The term sensitivity has been used in population ecology since late 1960s (Demetrius, 1969), representing the sensitivity of population growth rate to matrix elements (derived from vital rates) expressed in terms of left and right eigenvectors of the MPM (i.e., reproductive value and stable stage distribution, respectively).

During the following two decades, several papers extended and improved the usage of sensitivities and elasticities, always focusing on a first‐order effect of demographic process perturbation on fitness (Figure 2, green arrow). Caswell's (1996) publication on the second‐order derivatives of population growth rate widened the scope from questions restricted by the linearity assumption to also ecological and evolutionary questions that could account for the nonlinear relationship between the fitness function and the vital rates (Brooks et al., 2005; Carslake et al., 2008; Stott et al., 2012) (Figure 2, yellow arrow).

The move to report integrated linear (elasticity) and nonlinear (second‐order) effects of fitness (Figure 2, green‐yellow arrow) stems from the long‐known recognition that the demographic responses are often nonlinear, with thresholds (Folke et al., 2004; Stephens et al., 1999), saturation (Holling, 1965), or trade‐offs (van Tienderen, 1995). Also, the importance of identifying nonlinear effects of perturbations on demographic processes on fitness has been long discussed (Carslake et al., 2008, 2009; Stott et al., 2012; Barraquand & Yoccoz, 2013; Shyu & Caswell, 2014; Jaggi et al., 2026). Considering nonlinear fitness effects alongside linear elasticity can thus contribute crucial information: While elasticity indicates whether λ increases or decreases with a certain vital rate (Caswell, 2001; de Kroon, Van Groenendael, and Ehrlen, 2000; Ebert, 1999), the S‐elasticity answers a deeper biological question of whether λ increases at an *accelerating* or a *diminishing* rate. An answer to the latter question represents an information needed to understand trade‐offs, environmental stress responses and limits to local adaptation, by answering not only *whether* a demographic process matters, but *how* it matters.

Here we implement the S‐elasticity (where “S” stands for second order and is denoted by ${\hat{e}}_{\mathrm{ij},\mathrm{kl}}$), a second‐order elasticity measure with the ability to scale the nonlinear fitness responses to be dimensionally consistent with the first‐order elasticities. Our method recovers the same information as classical approaches (Caswell, 1996; Kajin et al., 2023; Shyu & Caswell, 2014) but avoids Kronecker products, keeps the computation in more familiar matrix space, and allows a straightforward implementation. The S‐elasticity method uses eigen‐perturbation and transient‐response matrix (Jaggi et al., 2026), which expresses the Hessian (i.e., complete second‐order derivative matrix) in terms of familiar demographic objects like stable age distribution, the reproductive value and the transient phases of population's dynamics. The S‐elasticity reveals element‐wise curvature (concave/convex) of the mean individual fitness and mixed‐term diagnostics for pairs of matrix elements in a format that is easy to interpret: signs of the S‐elasticity classify local concavity or convexity; magnitudes locate where curvature is negligible versus influential relative to elasticity; and mixed terms diagnose interactions between MPM elements.

We detail some key mathematical properties of the S‐elasticity and show how this new measure brings several key biological interpretations by using the same data set for which classical second derivative of population growth rate had been reported (Kajin et al., 2023). We show that because S‐elasticities are scaled, they can be linked to a measure known from economics as the marginal rate of substitution (Jones & Tuljapurkar, 2015). The latter connection yields unique insights into a life cycle by explicitly linking costly trade‐offs (where change in one vital rate is costly for the other one) to the type of nonlinear response (convex/concave) that said trade‐offs provoke on the mean individual fitness. As such, the S‐elasticity is an important complement to the toolbox of ecologists and evolutionary biologists examining the dynamics of population responses to increased environmental variation.

## ELASTICITY AND S‐ELASTICITY

### Defining S‐elasticity

In a discrete time and a constant environment setting, we can describe the dynamics of a structured population by a population matrix A. Here, the population is structured into discrete stages that are represented by age classes, developmental stages, and/or discrete‐size classes (Caswell, 2001). The entries $a_{\mathrm{ij}}$ of the matrix A are matrix elements composed of vital rates (Zuidema & Franco, 2001), the rates of transitions (from *i* to *j*) between ages, stages, and/or sizes (survival, development, etc.) and per capita contributions (a/sexual reproduction). When the matrix A is primitive and irreducible (meaning that no stage is permanently isolated and that all stages are eventually connected by the sequence of matrix transitions, ensuring a single $\lambda_{0}$ and stable structure for elasticity analyses), A has a dominant eigenvalue $\lambda_{0}$ with associated right $u_{0}$ and a left eigenvector $v_{0}$. In the context of elasticities/sensitivities, these eigenvectors are typically normalized so that their scalar product $<v_{0},u_{0}>=1$. When averaged across the population, the dominant eigenvalue of ***A*** represents the mean individual fitness in the population ($\lambda_{0}$) described by A (Caswell, 2001).

The behavior of the mean individual fitness in a structured population can be studied using a matrix A. Say that the environment or evolutionary pressures change a matrix element $a_{\mathrm{ij}}$ by a small proportion so that $a_{\mathrm{ij}}$ becomes $a_{\mathrm{ij}}\left(1+\varepsilon \right)$. The elasticity of $\lambda_{0}$ to $a_{\mathrm{ij}}$ is the proportional *linear* change in $\lambda_{0}$ divided by the proportional change in $a_{\mathrm{ij}}$ (Caswell, 1978; de Kroon et al., 1986). Because elasticities of matrix elements sum to unity across A (Ebert, 1999; de Kroon, Van Groenendael, and Ehrlen, 2000; see also section Sum of S‐elasticity below), they allow for a comparison of the relative contributions of each matrix element to the overall population growth rate across populations (Enright et al., 1995) and species (Franco & Silvertown, 2004). However, the sum‐to‐unity rule implies that elasticities of $\lambda_{0}$ to matrix elements within MPM are not mathematically independent (Shea et al., 1994). When two matrix elements change, the sum‐to‐unity constraint means that increasing the relative contribution of one element necessarily reduces the share attributable to other element (Doak et al., 2005; Jones & Tuljapurkar, 2015; Roff, 2002; van Tienderen, 2000). Interpreting fitness responses only through this linear, first‐order lens can therefore miss important behavior when elements change together. When two matrix elements, say $a_{\mathrm{ij}}$ and $a_{\mathrm{kl}}$, are perturbed simultaneously, the corresponding change in mean individual fitness $\lambda_{0}$ is composed of both elements' linear (elasticity) terms plus a mixed second‐order term, determining whether the joint response is amplified or damped, that is, whether the local fitness surface is concave or convex in that pair. To quantify these curvature properties, we use S‐elasticities, where we further divide the nonlinear second‐order term into two cases:

1. For a single element, we describe the self S‐elasticity as

(1) $${\hat{e}}_{\mathrm{ij},\mathrm{ij}}=\frac{{a_{\mathrm{ij}}}^{2}}{{\lambda_{0}}^{2}}\frac{\partial^{2}\lambda_{0}}{\partial {a_{\mathrm{ij}}}^{2}},$$

which classifies whether λ_0_ is locally concave (${\hat{e}}_{\mathrm{ij},\mathrm{ij}}<0$) or convex (${\hat{e}}_{\mathrm{ij},\mathrm{ij}}>0$) with respect to $a_{\mathrm{ij}}$.

2 For a pair of elements $a_{\mathrm{ij}}$ and $a_{\mathrm{kl}}$, we use the mixed S‐elasticity defined as

(2) $${\hat{e}}_{\mathrm{ij},\mathrm{kl}}=\frac{a_{\mathrm{ij}}a_{\mathrm{kl}}}{{\lambda_{0}}^{2}}\frac{\partial^{2}\lambda_{0}}{\partial a_{\mathrm{ij}}{\mathrm{\partial a}}_{\mathrm{kl}}},$$

which displays the nonlinear interaction between the two elements (antagonism if ${\hat{e}}_{\mathrm{ij},\mathrm{kl}}<0$, synergy if ${\hat{e}}_{\mathrm{ij},\mathrm{kl}}>0$).

Equation (1) implies that the sign of the self S‐elasticity is equal to the sign of the classical second derivative. Under standard stage‐structured parametrizations with nonzero stasis $a_{\mathrm{ii}}$, stasis entries have positive second‐order effects, whereas advancing survival/growth $a_{1+1,i}$ have negative second‐order effects (Carslake et al., 2008). However, in a Leslie‐type MPM, where all $a_{\mathrm{ii}}=0$, the stasis constraint is inapplicable; only the advancing survival/growth prediction (negative curvature) remains, while fertility has no universal sign.

When two matrix elements are simultaneously perturbed by a small, one‐time pulse disturbance (e.g., a drought, a single freezing spring, management action), the nonlinear part of the response can only be captured by the second derivative of $\lambda_{0}$ with respect to those two elements (Jaggi et al., 2026). Linking the curvature to the population's transient response using the eigen‐perturbation approach, the mixed second derivative can also be written as

(3) $$\frac{\partial^{2}\lambda_{0}}{\partial a_{\mathrm{ij}}{\mathrm{\partial a}}_{\mathrm{kl}}}=s_{\mathrm{il}}J_{0,\mathrm{jk}}+s_{\mathrm{kj}}J_{0,\mathrm{li}},$$

where the term $s_{\mathrm{ij}}$ corresponds to the sensitivity of population growth rate with respect to $a_{\mathrm{ij}}$

(4) $$s_{\mathrm{ij}}=\frac{\partial \lambda_{0}}{\partial a_{\mathrm{ij}}},$$

and the entries of *J*_0_ quantify how a pulse in a single transition propagates through the stage structure before the population returns toward its stable state (i.e., the transient response). The *J*_0_ matrix (transient response matrix, TRM) (entries *J*_0,*jk*_, *J*_0,*li*_ in Equation 3) essentially captures the cumulative impact of these small changes over time, linking the immediate, short‐term responses (transient dynamics) to the long‐term, stable state of the population. The pulse/press link (press disturbances sensu Menge & Sutherland, 1976) and the *J*_0_ operator are introduced in Jaggi et al. (2026), which shows how second derivatives of λ_0_ relate to transient dynamics. Standard definitions of sensitivities and elasticities are given in Caswell (2001), where elasticity $e_{\mathrm{ij}}$ of fitness (Caswell, 2001; Ebert, 1999; Jaggi et al., 2026) is the proportional change that results in λ_0_,

(5) $$e_{\mathrm{ij}}=\frac{a_{\mathrm{ij}}}{\lambda_{0}}\frac{\partial \lambda_{0}}{\partial a_{\mathrm{ij}}}.$$

### Nonlinearity in fitness

S‐elasticities describe the nonlinear response in mean individual fitness in the population to small, one‐off pulse perturbations in matrix elements (Equation 3). A change in the matrix element $a_{\mathrm{ij}}$ by a small proportion $\epsilon_{\mathrm{ij}}$ would cause mean individual fitness to change from λ_0_ to $\lambda^{\prime }$ such that

(6) $$\lambda^{\prime }=\lambda_{0}+\lambda_{0}\sum_{\left\{\mathrm{ij}\right\}}\epsilon_{\mathrm{ij}}e_{\mathrm{ij}}+\frac{1}{2}{\lambda_{0}}^{2}\sum_{\left\{\mathrm{ij},\mathrm{kl}\right\}}\epsilon_{\mathrm{ij}}\epsilon_{\mathrm{kl}}{\hat{e}}_{\mathrm{ij},\mathrm{kl}}+\mathrm{Rem},$$

where the remainder *Rem* is of cubic order in the largest of the fractions $\epsilon_{\mathrm{ij}}$. Equation 6 can be examined by observing the linear and nonlinear terms in $\lambda^{\prime }$. The linear term is based on elasticities ($e_{\mathrm{ij}}$, Equation 5), while the nonlinear term is based on the newly described self S‐elasticities for single elements (${\hat{e}}_{\mathrm{ij},\mathrm{ij}}$, Equation 1) and mixed S‐elasticities for pairs of elements (${\hat{e}}_{\mathrm{ij},\mathrm{kl}}$, Equation 2). To assess the relative impact of the nonlinear term compared to the linear term we can calculate the ratio of the S‐elasticity to the elasticity for: (1) single matrix element as $\frac{{\hat{e}}_{\mathrm{ij},\mathrm{ij}}}{e_{\mathrm{ij}}}$ and (2) a pair of matrix elements as $\frac{2{\hat{e}}_{\mathrm{ij},\mathrm{kl}}}{e_{\mathrm{ij}}+e_{\mathrm{kl}}}$. A high (positive or negative) S‐elasticity/elasticity ratio indicates that the nonlinear term is stronger relative to the linear term in the fitness response when either $a_{\mathrm{ij}}$ or both—$a_{\mathrm{ij}}$ and $a_{\mathrm{kl}}$—undergo small proportional changes. The sign of the S‐elasticity/elasticity ratio for a pair of matrix elements—involved in a biologically feasible trade‐off—further indicates whether the mean individual fitness is at peak of a concave surface (S‐elasticity/elasticity ratio <0), or at the bottom of a convex surface (S‐elasticity/elasticity ratio >0).

In a MPM A, vital rate trade‐offs (Roff, 2002) are reflected by negative correlations between two lower level matrix element parameters (Zuidema & Franco, 2001). If two matrix elements in A, say $a_{\mathrm{ij}}$ and $a_{\mathrm{kl}}$, are involved in a trade‐off, any perturbation in $a_{\mathrm{ij}}$ will result in $a_{\mathrm{kl}}$ being changed simultaneously; thus, the elasticities and the S‐elasticities of $a_{\mathrm{kl}}$ will be influenced, even if $a_{\mathrm{kl}}$ itself was not perturbed directly. Depending on the nature of the trade‐off between $a_{\mathrm{ij}}$ and $a_{\mathrm{kl}}$, the hypothetical proportional change in $a_{\mathrm{ij}}$ required to offset a proportional change in $a_{\mathrm{kl}}$—while maintaining fitness—can be either costly or not (Jones & Tuljapurkar, 2015). A large value of the ratio between the elasticities of $a_{\mathrm{ij}}$ and $a_{\mathrm{kl}}$ ($K=-\frac{e_{\mathrm{ij}}}{e_{\mathrm{kl}}}$, the *K*‐ratio, hereafter) characterizes costly trade‐offs, where compensating for a small proportional decrease in $a_{\mathrm{ij}}$ requires $a_{\mathrm{kl}}$ to increase by a large proportion, indicating steep constraint and weak substitutability between the two demographic processes (Jones & Tuljapurkar, 2015). Conversely, a small *K*‐ratio suggests that demographic processes can more easily compensate for each other, indicating weak constraint and stronger substitutability.

Suppose mean individual fitness is at an optimum before a change in two demographic processes. If fitness is at optimum (top of a hill on a concave fitness surface), the nonlinear term of the change in fitness is negative. For the nonlinear term of the change in fitness to be negative, the following inequality must hold true for any feasible trade‐off:

(7) $$0>\left[K^{2}{\hat{e}}_{\mathrm{kl},\mathrm{kl}}+2K\;{\hat{e}}_{\mathrm{kl},\mathrm{ij}}+{\hat{e}}_{\mathrm{ij},\mathrm{ij}}\right].$$

In this sense, we are looking for those pairs of demographic processes that are involved in costly trade‐offs and use the S‐elasticities to see whether the inequality holds or not. If the inequality holds, this means that the population is at a local peak in fitness for the said pair of demographic processes. However, if the inequality does not hold, the mean individual fitness in the population might be far from its local optimum and the population might be going through a transient phase (Stott et al., 2011, 2012).

### Sum of S‐elasticity

A second essential property of the S‐elasticity lies in its summed effect. Elasticities are often grouped for biological and mathematical reasons to examine and classify impacts on life history strategies (e.g., Franco & Silvertown, 2004; Takada & Kawai, 2020; Takada & Shefferson, 2018). In many cases, life history transitions share a process (e.g., growth), or an energy/mass flow (e.g., reproduction), or describe a common outcome (e.g., death). In such cases, elasticities are typically grouped together into vital rates (Franco & Silvertown, 2004; Grime, 1977; Silvertown et al., 1992; Takada & Kawai, 2020).

Another way of grouping life history transitions defined by a MPM A is by following a life cycle loop (van Groenendael et al., 1994). Complex life cycles can be composed of many simple pathways (loops), where one loop may represent early reproduction and another loop late reproduction (Wardle, 1998). By breaking down the life cycle of an organism into multiple constituent loops, we can identify life history strategies that most influence $\lambda_{0}$. Rather than focusing on a single demographic process, as classic elasticity analysis tends to do (e.g., Caswell, 2001; de Kroon, Van Groenendael, and Ehrlen, 2000; Ebert, 1999), the loop approach provides a comprehensive understanding of the paths with the highest characteristic loop elasticities—which are particularly important for the long‐term population growth rate.

Grouping S‐elasticities also bears biological meaning. If every demographic process $a_{\mathrm{ij}}$ of the matrix A changes by the same small proportion ε, then, the new fitness becomes $\lambda^{\prime }$ as per Equation (6). Another way of thinking about the new fitness value following a change by the same proportion in all $a_{\mathrm{ij}}$ values means that the MPM changes from A to $\left(1+\epsilon \right)$ ***A***. However, compared to the approach of changing individual demographic processes separately, the latter view simply means that mean individual fitness in population changes from $\lambda_{0}$ to:

(8) $$\lambda^{\prime }=\lambda_{0}+\epsilon \lambda_{0}.$$

Because Equations (6) and (8) are identical, the following must hold:

(9) $$\sum_{\mathrm{ij},\mathrm{kl}}{\hat{e}}_{\mathrm{ij},\mathrm{kl}}=0.$$

The S‐elasticities summing to zero leads to the S‐elasticities of different demographic processes not being independent of each other. Furthermore, if the Hessian (i.e., the complete matrix of all the second‐order derivatives) is not all 0 (trivial case), then the Hessian has at least one positive and one negative element (Sun & Sugie, 2019). The latter yields the known result that the usual first‐order elasticities of matrix elements sum to unity: $\sum_{\mathrm{ij}}e_{\mathrm{ij}}=1$ (de Kroon, Van Groenendael, and Ehrlen, 2000). The summative characteristics of elasticities and S‐elasticities bear intriguing evolutionary implications, as they depict the distribution of importance in either ecological or evolutionary terms among various demographic processes in sustaining the long‐term stability of population growth rates. The fact that the S‐elasticities also sum to zero highlights their usefulness for comparative demography, across species and populations, and within populations across time and treatments.

### Example: Elasticities and S‐elasticities for *Didelphis aurita*

To illustrate the insights that the S‐elasticity can provide, we show the elasticities and S‐elasticities for a species of mammal, the black‐eared opossum (*Didelphis aurita*, Marsupialia, Didelphidae), using the data available in Kajin et al. (2008) and Kajin et al. (2023). We chose this data set to allow for an explicit comparison between the performances of the newly described S‐elasticities and the classical second‐order derivative approach as described by Caswell (1996) and Shyu and Caswell (2014) and show the distinct new demographic information most transparently revealed by S‐elasticities.

The MPMs were composed as follows: in the sub‐diagonal $a_{21}$ = survival of weaned, $a_{32}$ = survival of subadults, $a_{33}$ = survival of adults; $a_{12}$ = fertility of subadults (i.e., number of offspring produced by subadults × survival of pouch‐young inside the marsupium), $a_{13}$ = fertility of adults (i.e., number of offspring produced by adults × survival of pouch‐young inside the marsupium), $a_{14}$ = fertility of aged females (i.e., number of offspring produced by aged females × survival of pouch‐young inside the marsupium). We obtained the average matrix (by calculating the element‐by‐element arithmetic mean) of five MPMs, excluding atypical lifetables where λ_0_ < 1, to keep the demography generally representative.

To show the joint dynamics of the elasticity and the S‐elasticity, we calculated the population growth rate ($\lambda_{0}$), elasticities ($e_{\mathrm{ij}}$), S‐elasticities (${\hat{e}}_{\mathrm{ij},\mathrm{ij}}$ and ${\hat{e}}_{\mathrm{ij},\mathrm{kl}}$), the S‐elasticity/elasticity ratios ($\frac{{\hat{e}}_{\mathrm{ij},\mathrm{ij}}}{e_{\mathrm{ij}}}$ and $\frac{2{\hat{e}}_{\mathrm{ij},\mathrm{kl}}}{e_{\mathrm{ij}}+e_{\mathrm{kl}}}$), and the *K* ratio for pairs of matrix elements as described above using package *popbio* (Stubben et al., 2020) in RStudio (Posit team, 2024).

## RESULTS

**FIGURE 3:**
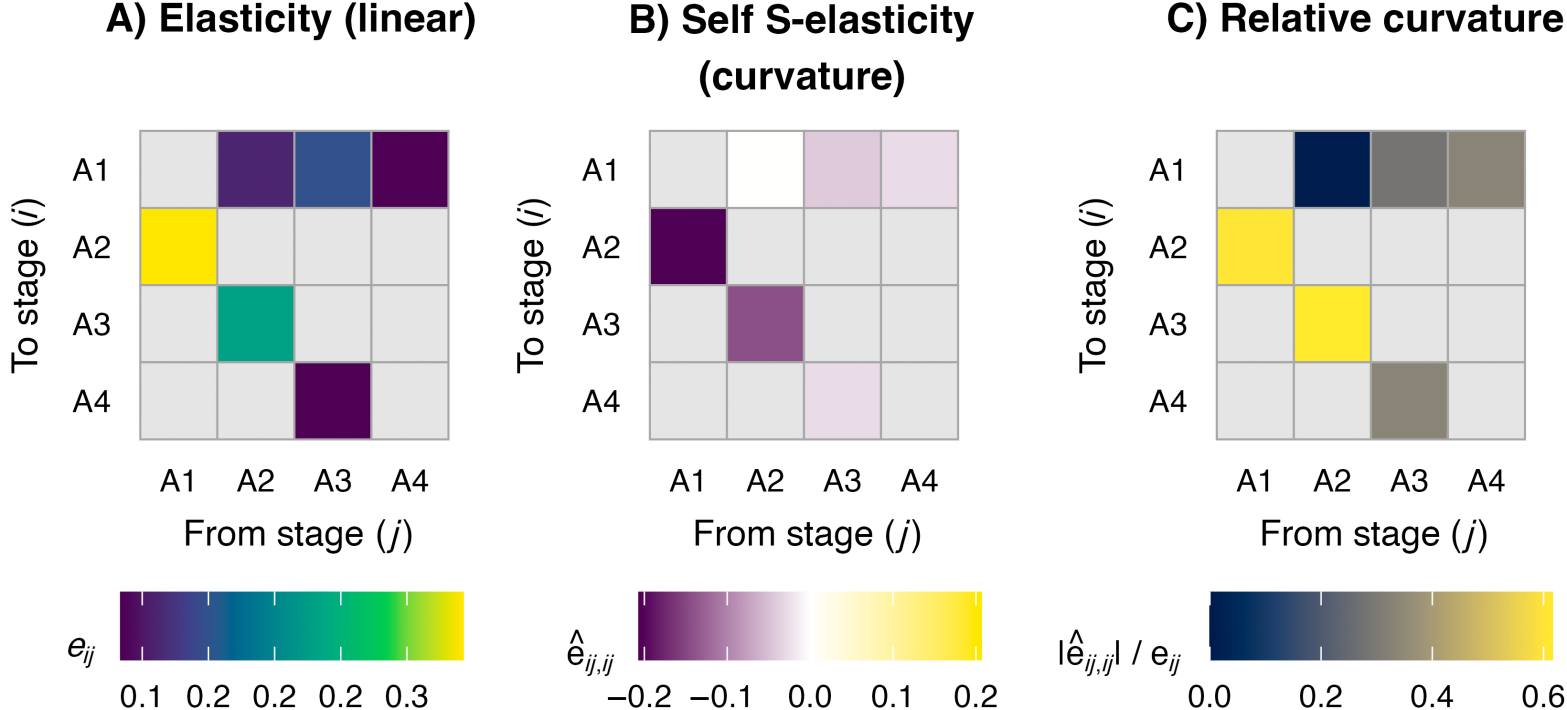
Elasticity, S‐elasticity, and relative curvature of mean individual fitness for a population of black‐eared opossum *Didelphis aurita* (Marsupialia, Didelphidae). Shown are the matrix population model (MPM) results for the average 4 × 4 projection matrix (based on vital rate data from Kajin et al. 2008 and Kajin et al., 2023). Panels show (A) elasticity $e_{\mathrm{ij}}$, the proportional first‐order change in population growth rate $\lambda_{0}$ in response to proportional change in matrix element $a_{\mathrm{ij}}$; (B) self S‐elasticity ${\hat{e}}_{\mathrm{ij},\mathrm{ij}}$, the second‐order (curvature) effect of perturbing a single matrix element $a_{\mathrm{ij}}$ on $\lambda_{0}$, where positive values indicate local convexity and negative values local concavity of the fitness; and (C) relative curvature $|{\hat{e}}_{\mathrm{ij},\mathrm{ij}}|/e_{\mathrm{ij}}$, the magnitude of curvature relative to the linear effect. Tiles corresponding to structural zeros in the MPM are masked (gray). Stage ordering and tile placement follow explicit element indexing (rows = “to” stage i, columns = “from” stage j).

The mean individual fitness of the population of *D. aurita* was near 1 ($\lambda_{0}=0.9799$) implying near‐stationary long‐term dynamics in the absence of density dependence and environmental stochasticity. Element‐wise elasticities $e_{\mathrm{ij}}$ mapped across the life cycle (Figure 3A) identify the demographic processes with larges linear leverage on λ_0_ and reveal the expected dominance of a small set of demographic processes.

Matrix element $a_{21}$ (surviving from stage 1 to stage 2, namely, survival of the weaned) displayed the highest value of first‐order elasticity, followed by matrix element $a_{32}$ (surviving from stage 2 to Stage 3, namely, survival of the subadult). Survival of the weaned opossums clearly dominates the importance over mean individual fitness (Figure 3A). Nonetheless, the self S‐elasticities for the same two demographic processes, namely, survival of the weaned and survival of subadults, evidence that $\lambda_{0}$ responds not only intensively, but with diminishing force, since concavity was detected by ${\hat{e}}_{\mathrm{ij},\mathrm{ij}}<0$ (Figure 3B). Because our MPM has no stasis entries (all diagonal $a_{\mathrm{ii}}=0$), the only general sign constraint that applies is the negative curvature for advancing survival/growth; this matches our estimates, whereas fertility shows mixed behavior.

The relative curvature $\frac{\left|\;,{\hat{e}}_{\mathrm{ij},\mathrm{ij}}\right|}{e_{\mathrm{ij}}}$ (Figure 3C) shows that concavity is not negligible for the survival of the weaned and survival of subadults ($a_{21}$ and $a_{32}$, respectively). Both these demographic processes, with appreciable elasticity, also exhibit nonzero relative curvature (Figure 3c), indicating that linear rankings alone do not fully describe local fitness responses for the most important demographic processes as identified by first‐order elasticities. Thus, even for those demographic processes where $\frac{\left|\;,{\hat{e}}_{\mathrm{ij},\mathrm{ij}}\right|}{e_{\mathrm{ij}}}<1$, this ratio identifies which demographic processes operate on a concave/convex fitness surface (diminishing/accelerating returns), and where the fitness surface is nearly linear. The $\frac{\left|\;,{\hat{e}}_{\mathrm{ij},\mathrm{ij}}\right|}{e_{\mathrm{ij}}}$ reveals that linear elasticity is not invalid but incomplete. It remains accurate in sign, but not in slope, that is, the rate at which $\lambda_{0}$ changes, which is not constant. The self S‐elasticity quantifies this changing slope. Because $a_{21}$ and $a_{32}$, show ${\hat{e}}_{\mathrm{ij},\mathrm{ij}}<0$ (Figure 3B) with nonzero relative curvature (Figure 3C), the elasticity‐based linear approximation overestimates gains from increasing these survivals and underestimates losses from decreasing them.

**FIGURE 4:**
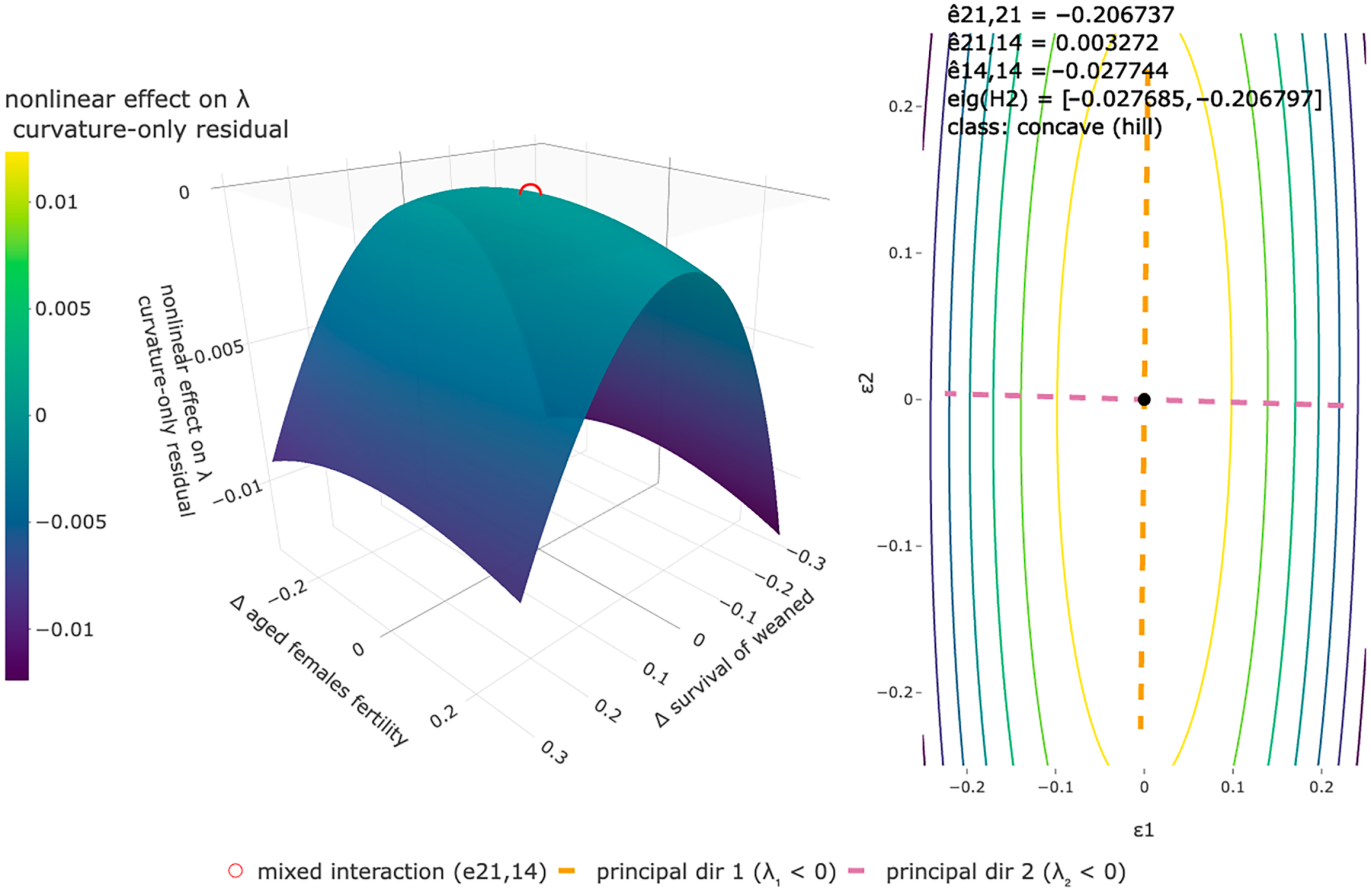
Curvature‐only nonlinear effect of jointly perturbing survival of weaned and aged female fertility in *Didelphis aurita* (Marsupialia, Didelphidae). Second‐order diagnostics for the average 4 × 4 matrix population model (MPM) when jointly perturbing $a_{21}$(survival of weaned) and $a_{14}$(aged females' fertility). Left/center panel: 3D surface of the curvature‐only residual, $Z_{\text{curv}}=\lambda_{\text{exact}}-\lambda_{\text{linear}}$, across proportional perturbations $\varepsilon_{1}$(*x* axis, Δ survival of weaned) and $\varepsilon_{2}$(*y* axis, Δ aged females' fertility). Colors indicate the nonlinear contribution to $\lambda_{0}$ after removing the first‐order (elasticity) plane; values near zero (green) indicate weak nonlinearity, warmer/cooler colors indicate stronger positive/negative curvature effects. The open red marker at the origin encodes the sign of the mixed S‐elasticity ${\hat{e}}_{21,14}$(red = positive; size $\propto |{\hat{e}}_{21,14}|$). Right panel: Contour map of the quadratic form $q\left(\varepsilon_{1},\varepsilon_{2}\right)$ derived from the 2 × 2 Hessian of λ for this pair; dashed lines show the principal curvature directions (eigenvectors), with line style reflecting the sign of the corresponding eigenvalues (here both eigenvalues <0, indicating local concavity/a “hill”). Together, these panels isolate and visualize how nonlinear interactions between survival of weaned and aged females' fertility amplify or counteract linear expectations around the baseline MPM. Structural zeros are not shown because the analysis is restricted to this biologically interpretable pair.

To show an example of the mixed S‐elasticities, we searched for a pair of demographic processes involved in a costly trade‐off (with weak substitutability). We found that compensating for a small proportional decrease in $a_{21}$ (survival of weaned), requires $a_{14}$ (aged females' fertility) to increase by a large proportion. This is so because survival of weaned and aged females' fertility returned the highest values of the *K*‐ratio ($K_{21,14}=-4.096$, results not shown, but available in provided code). For the trade‐off between survival of weaned and aged females' fertility, we display the concave effect on mean individual fitness in Figure 4.

The nonlinear effect on $\lambda_{0}$ is represented by the curvature‐only residual, Z (Figure 4). The curvature‐only residual displaying the concave surface depicts $Z=\lambda_{\text{exact}}-\lambda_{\text{linear}}$, where λ_exact_ is the true dominant eigenvalue of the perturbed matrix [$\lambda_{\text{exact}}\left(\varepsilon_{1},\varepsilon_{2}\right)=\lambda (A|\;a_{\mathrm{ij}}\to a_{\mathrm{ij}}\left(1+\varepsilon_{1}\right),a_{\mathrm{kl}}\to a_{\mathrm{kl}}\left(1+\varepsilon_{2}\right)$)], and $\lambda_{\text{linear}}$ is the first‐order (linear) prediction from classic elasticity [$\lambda_{\text{linear}}\left(\varepsilon_{1},\varepsilon_{2}\right)=\lambda_{0}+\lambda_{0}\left(\varepsilon_{1}e_{21}+\varepsilon_{2}\;e_{14}\right)$] (Figure 4, left panel). *Z* therefore depicts only the nonlinear part of the mean individual fitness response. The full Taylor expansion is given in Equation (6), and when we compute Z, we remove the first‐order term and what remains is the quadratic curvature (approximately corresponding to the quadratic term in Equation 6 when ε remains small). The concave surface for the pair survival of weaned and aged females' fertility forms a “hill” shaped dome around the baseline, with Z≈0 at the origin but as proportional changes move further from zero, the negative residuals increase (Figure 4, left panel). This pattern confirms diminishing returns to perturbations in either demographic process when considered near the current life history state.

The contour map of the quadratic form (Figure 4, right panel) shows elliptical sets with both principal curvatures (eigenvalues) negative, offering also geometric evidence of concavity. Ellipses are elongated, and the curvature is stronger alongside a direction mostly aligned with the change in survival of weaned ($a_{21}$). This means that any changes in survival of weaned opossums reduces mean individual fitness faster than equal‐sized changes in aged females' fertility. The mixed S‐elasticity at the baseline is positive (Figure 4, left panel, red open circle at the origin), evidencing that the two demographic processes interact nonlinearly rather than additively. Together, these diagnostics indicate that, for this pair of demographic processes, $\lambda_{0}$ is at local peak and that joint changes away from the current values are penalized to a second order, with this penalty being stronger along the survival‐of‐weaned direction, as compared to aged females' fertility.

**FIGURE 5:**
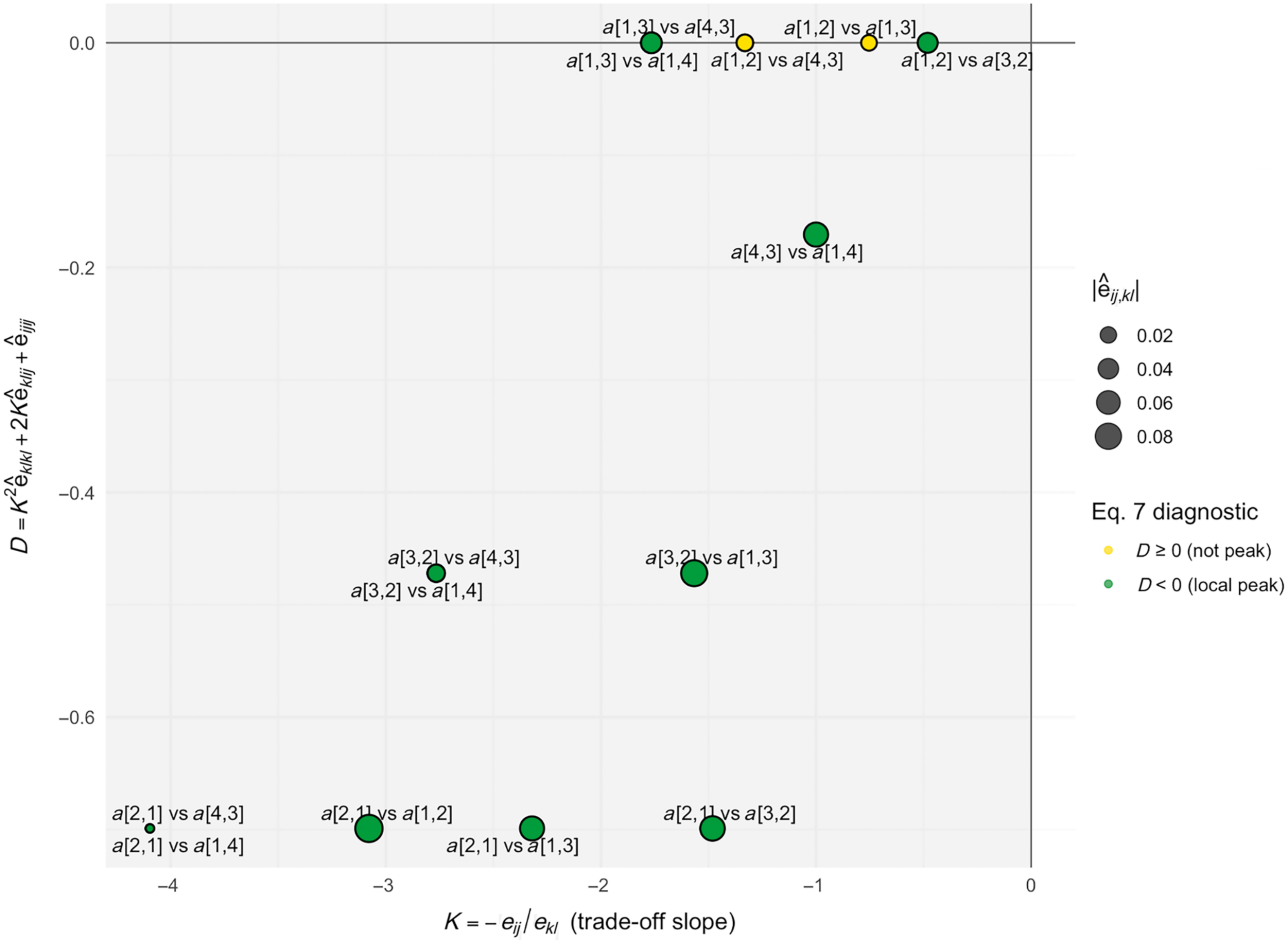
Curvature (Equation 7) and substitutability between pairs of demographic processes in *Didelphis aurita* (Marsupialia, Didelphidae). Each point is a feasible pair of demographic processes (represented by matrix elements *a*_*ij*_ and *a*_*kl*_ from the average 4 × 4 matrix population model. The *x* axis shows the trade‐off slope $K=-e_{\mathrm{ij}}/e_{\mathrm{kl}}$ where large $\left|K\right|$ indicates low substitutability. Our use of “trade‐off” is diagnostic: It specifies a constraint direction along which we test curvature (Equation 7), rather than inferring empirical covariation among vital rates. The *y* axis shows the Equation 7 diagnostic, which tests the local curvature of mean individual fitness along that trade‐off direction: green points (D<0) indicate a local peak (concave “hilltop”); yellow points (D≥0) indicate directions that are flat or convex (not a peak). Point size is proportional to the magnitude of the mixed S‐elasticity, the strength of the nonlinear interaction between the two elements. Labels identify the element pair in matrix notation $a_{\mathrm{ij}}$ (row *i* = “to” stage; column *j* = “from” stage). The vertical line marks K=0; the shaded half highlights the typical K<0 region when both elasticities are positive (because $K=-e_{\mathrm{ij}}/e_{\mathrm{kl}}$).

We further depict the performance of the mixed S‐elasticities in combination with the *K*‐ratio. Our Equation (7) diagnostic (Figure 5) evaluates curvature along pairs of demographic processes. For the pair survival of weaned ($a_{21}$) and aged females' fertility ($a_{14}$), we obtain D<0 and $\left|K\right|\gg 1$ (Figure 5). This indicates a local fitness peak (concavity) alongside low substitutability—small trade‐off shifts are expected to decrease $\lambda_{0}$ and are hard to compensate. In contrast, pairs in the upper right corner of the plot (dominated by fertility elements $a_{1x}$) cluster near D≈0 or >0 with smaller $\left|K\right|$ values, indicating that along those trade‐off directions the fitness surface is flat or convex (i.e., not a local peak), and the demographic processes are more easily exchanged among each other. In other words, more scope for variation with a weaker nonlinear penalty on λ_*0*_ is allowed in pairs with small $\left|K\right|$ values. Where, for such pairs, the mixed S‐elasticity is small, curvature is weak, implying a tolerant iso‐fitness direction in which modest changes in the pair have limited nonlinear impact on $\lambda_{0}$.

## DISCUSSION

S‐elasticities reveal the sign and magnitude of local curvature in mean individual fitness that elasticity cannot detect, regardless of magnitude. The S‐elasticities show where increases in demographic processes yield diminishing returns (concavity) or accelerating returns (convexity), and where pairs of demographic processes interact synergistically or antagonistically. In our case study, this exposes when linear rankings are adequate (near‐zero relative curvature) and when they over/under‐predict the consequences of, for example, management actions (Figure 3C), and it identifies hilltop trade‐offs where feasible substitutions are both costly and fitness‐decreasing (Figures 4 and 5). Unlike earlier second‐derivative approaches expressed on absolute scales (Caswell, 1996; Kajin et al., 2023; Shyu & Caswell, 2014), the S‐elasticities provide proportional second‐order measures in matrix element space, sum to zero across elements, and, via the *K*‐ratio and Equation (7), directly test curvature along feasible trade‐off directions.

A fundamental challenge in extending perturbation analysis from linear to nonlinear effects is the rapid expansion of dimensionality. This means that a 4 × 4 MPM will result in a single Hessian matrix of dimensions 16 × 16, containing 256 s‐order interaction terms. While the fitness landscape is a single surface, its curvature is described by the simultaneous interactions among all simultaneously varying demographic processes. For the *D. aurita* life cycle, which comprises multiple nonzero transitions, the complete fitness landscape technically represents a multidimensional parameter space because the principal directions of curvature (eigenvectors of the Hessian) extend across all demographic processes simultaneously. Consequently, one can overcome this complexity by rigorously examining meaningful interactions. We reduced the dimensionality in Figure 4 by slicing the fitness surface along a plane defined by a specific, biologically relevant pair of demographic processes identified by large *K*‐ratio (Jones & Tuljapurkar, 2015). By isolating the ‘costly trade‐off’ between survival of weaned individuals and aged fertility, we transform an abstract multidimensional interaction into a discernible landscape, allowing us to visually diagnose the local stability properties specific to this key life history constraint.

The S‐elasticity connects the mean individual fitness curvature to biologically feasible trade‐offs via the *K*‐ratio (Jones & Tuljapurkar, 2015) (Figures 4 and 5). The Equation 7 diagnostic tests for peak‐like curvature along feasible biological constraint directions—capabilities that make the S‐elasticity decision‐relevant. In addition, by expressing the Hessian with an eigen‐perturbation and transient‐response operator *J*_0_ (Jaggi et al., 2026), the S‐elasticity links nonlinear (second‐order) effects to transient dynamics, a connection not available before. The transient‐response matrix *J*_*0*_ provides the bridge from fitness curvature to life history biology: Each S‐elasticity is a weighted account of how a brief pulse in one transition flows through life cycle stages before the population bounds back toward the stable state. Biologically, entries of *J*_0_ quantify how juveniles progress to subadults and adults, how fecundity pulses feed back to the juvenile stage, and how long individuals linger in (or quickly leave) each stage. Thus, while *J*_0_ stores the species' life cycle features, the S‐elasticities inherit a direct biological meaning: positive mixed terms signal synergistic pathways (life cycle flows that raise the leverage of the other demographic process), whereas negative mixed terms signal antagonistic pathways (flows that dampen the other demographic process's influence). That is exactly why, in *D. aurita*, the pair survival of weaned and aged females' fertility yields a concave “hill” in Figure 4 and *D* < 0 in Figure 5: The early survival pathway is a bottleneck, and *J*_0_ assigns large weight to flows that pass through it, so small co‐perturbations along that costly trade‐off quickly encounter diminishing returns.

The mixed S‐elasticity for *D. aurita* revealed additional life cycle information: Huge increases in aged females' fertility would be required to compensate for only a small decrease in survival of weaned while maintaining mean individual fitness (Figures 4 and 5). Such a signature, revealed by the mixed S‐elasticities of *D. aurita* (Figures 4 and 5), is compatible with mammalian senescence (Hamilton, 1966), where the importance of aged females' fertility contribution to the population growth rate is considerably lower as compared to the contributions of earlier age classes. Namely, the adult female fertility ($a_{13}$) and survival of weaned ($a_{21}$) are bound by a much less steep trade‐off slope, meaning their substitution is not as costly and adult fertility can more easily compensate for survival of weaned (Figure 5), while fitness is on a local peak for this pair of demographic processes.

What Figure 5 also reveals is that both aged females' fertility ($a_{14}$) and survival into the aged class ($a_{43}$) lack the power to compensate for even smallest decreases in survival of weaned (for being characterized by high *K*‐ratios). At the same time, mean individual fitness is at local peak for the two said pairs (pair $a_{21}$ and $a_{14}$, as well as $a_{21}$ and $a_{43}$ pair). Noticeably, the mean individual fitness is at local peak for all the pairs where survival of weaned ($a_{21}$) is involved (Figure 5, negative values for *D*), which designates this demographic parameter a special role inside the opossums life cycle: Any changes in survival of weaned is difficult to compensate and will result in dislocation from the local peak leading the population into transient dynamic phases. If survival of weaned needed to be compensated, changing the survival of subadults into adults (*a*_3,2_) would provide the fastest response; however, the response is still characterized as a costly one (Figure 5, $\left|K\right|<1$). Trade‐offs among other than aged females' fertility related demographic processes are characterized by less costly substitutions and localized away from local fitness peaks, allowing these demographic processes to vary with less consequences on the long‐term population growth rate.

Depending on the situation, it might be more realistic to prevent demographic process from being perturbed than attempting to increase their mean values (Andrello et al., 2020; Ramula et al., 2008). Therefore, by studying a higher (i.e., second‐) order effect of perturbations on fitness using S‐elasticity, an alternative potential of an effective fitness optimum is revealed. Such a combined application of both first‐ and second‐order effects of demographic process perturbation on mean individual fitness in a population holds a potential for population ecology (Santos et al., 2026). This combined application indicates that elasticities can offer more valuable insights for species management, maximizing yield, or conservation efforts than simply identifying the demographic process with the highest importance for the population growth rate (Mills et al., 1999; Rojas‐Sandoval & Meléndez‐Ackerman, 2013; Wisdom et al., 2000).

Examining the nonlinear mean individual fitness responses to perturbations in demographic processes can indicate a shift from concave to convex selection as environmental conditions change. Fitness shifts from concave to convex (or vice versa) are worth considering in the light of global climate change and habitat modification (Albaladejo‐Robles et al., 2022; Paniw et al., 2018). This shift of selection type aligns with the broader eco‐evolutionary framework, which proposes that rapid environmental changes necessitate equally dynamic and complex evolutionary responses (Gaillard et al., 2005; Shefferson & Salguero‐Gómez, 2015).

Additional important feature of studying a second‐order effect of perturbation on fitness is that it allows identifying the current stage of a studied population in terms of population stability (Farkas & Hagen, 2007). The introduced inequality (Equation 7, Figures 4 and 5) provides a diagnostic tool of whether a population is at a local peak in fitness or far from an optimum (going through a transient phase), a diagnosis that is of utmost importance when a strategy for management is being set up (Gifford et al., 2011; Jain et al., 2011; Rozen et al., 2008). The latter diagnosis combined with above detailed S‐elasticity features provide a significant improvement to our understanding of how natural plant and animal populations might strategize and optimize their strive toward environmental unpredictability.

Future research should expand on the above findings by applying S‐elasticity methodologies across various taxa and ecosystems to verify and expand upon these initial insights. Moreover, integrating long‐term field studies and controlled experimental designs will provide empirical support for a better understanding of the behavior of mean individual fitness, validating how nonlinear selection and fitness responses emerge in natural settings (Doherty et al., 2004; Rodríguez‐Caro et al., 2021). Through such integrative strategies, we hope to contribute toward a more nuanced understanding of population resilience and adaptability in an ever‐changing world to better anticipate and mitigate the impacts of rapid environmental change on biodiversity (Hamilton, 1966; Jaggi et al., 2026).

## CONFLICT OF INTEREST STATEMENT

All authors declare no conflicts of interest.

## Data Availability

Data and code (Kajin, 2026) are available in Figshare at https://doi.org/10.6084/m9.figshare.31281427.v1.
